# Learning lessons from cancer centers in low- and middle-income countries

**DOI:** 10.1186/1750-9378-8-44

**Published:** 2013-11-14

**Authors:** Brenda Kostelecky, Edward L Trimble, Kishor Bhatia

**Affiliations:** 1 Center for Global Health, National Cancer Institute, National Institutes of Health, 9609 Medical Center Drive, Rockville, MD 20850, USA; 2 Office of HIV and AIDS Malignancy, National Cancer Institute, National Institutes of Health, 9609 Medical Center Drive, Rockville, MD 20850, USA

## Abstract

Infectious Agents and Cancer is introducing a new section on Cancer Centers in Low- and Middle-Income Countries intended to provide the oncology community with detailed information about lessons learned in cancer control in resource-limited settings. The growing burden of cancer and the high rates of infection-related cancers in Low- and Middle-Income Countries (LMICs) argue for exploring the successes and challenges of cancer centers in low-resource settings. Detailed analyses are needed on how successful cancer centers have developed and managed such key components as strategic partnerships, trained cancer professionals, sustainable funding, appropriate technology, and research capacity. Many examples exist wherein local cancer centers have made significant progress and as such, the series will provide a platform to showcase detailed features of cancer institutes in LMICs and provide valuable information for those seeking to replicate successful models and to help invigorate efforts to build cancer capacity.

## 

Infectious Agents and Cancer is pleased to present a section entitled *Cancer Centers in Low- and Middle-Income Countries*. As low- and middle- income countries (LMICs) continue to face health challenges from infectious disease, they are concurrently challenged by the rising burden of non-communicable diseases including cancer. The International Agency for Research on Cancer (IARC) projects that by 2030, the annual number of new cancer cases worldwide will exceed 21 million and the number of cancer deaths will reach 13 million [1]. The World Health Organization (WHO) estimates that about 70% of all cancer deaths occur in LMICs and more than 20% of cancers in LMICs are directly attributable to infectious agents [2]. Infectious agents associated with cancers more frequently found in developing countries include Epstein-Barr virus (EBV), Kaposi sarcoma-associated herpesvirus/human herpesvirus 8 (KSHV/HHV8), human papillomavirus (HPV), hepatitis B and C viruses (HBV/HCV), human T-lymphotropic virus type 1 (HTLV-1), the bacterium *Helicobacter pylori*, and parasites *Opisthorchis viverrini* and *Schistosoma haematobium.* In Sub-Saharan Africa, the fraction of infection-related cancers is particularly high at 32.7% [3]. These numbers provide a compelling reason to explore successful strategies for enhancing cancer prevention and control capacity in LMICs. This enhanced capacity in LMICs includes strategic partnerships, well-trained professionals, appropriate technology, strengthened health systems, sustainable funding, and a solid research base to strengthen development of locally appropriate solutions in the fight against cancer.

Existing models of collaborative partnerships between a combination of low-, middle- and upper-income country cancer centers could provide useful pointers for improving cancer prevention and control capacity in low-resource settings. The challenges posed by the rising burden of cancer in Africa for instance, has already begun to influence new partnerships and expand previously existing partnerships between institutions globally. For instance, the long-term “twinning” program between Indiana University and Moi University in Kenya, AMPATH (Academic Model Providing Access to Healthcare), has built a strong foundation for health-related research and has put in place detailed standard operating procedures to ensure the continued success of the partnership [4]. Amongst the AMPATH program’s recent successes in Kenya in the field of cancer are training and mentoring of local oncology researchers [5] and nurses [6], creating oncology pharmacies [7], and evaluating chemotherapy outcomes for AIDS-associated Kaposi sarcoma [8]. Other examples of successful collaboration between U.S. and African institutions include the University of North Carolina’s (UNC) collaboration with the Kenya Medical Research Institute (KEMRI) and the Uganda Cancer Institute (UCI)/Hutchinson Cancer Center Alliance. The UNC/KEMRI collaboration has recently investigated HPV testing with self-collection of samples [9] and women’s attitudes toward HPV vaccine in Kenya [10]. The UCI/Hutchinson Cancer Center Alliance researchers have established that HIV plays a role in decreasing cancer survival in Uganda [11] and have investigated how gender influences Kaposi sarcoma presentation and outcomes [12]. Such collaborative partnerships can help catalyze a local institution’s capacity to provide preventive services, implement screening programs, deliver treatment and palliative care, procure quality cancer drugs, train allied health professionals in cancer care, strengthen cancer research on locally significant problems, support best practices in cancer care and research, develop healthcare informatics, and much more.

In addition to partnerships between institutions in high- and low-income countries, there is also a clear need to understand how institutes in LMICs can support one another in cancer prevention, control and research. With the increased strength of biological sciences in many developing countries [13], significant progress is certainly within reach and extending the benefits to cancer control will require integration of this growing capacity into cancer centers in LMICs. Partnerships between institutions within a country could ensure optimal use of resources by leveraging capacity from one disease setting (e.g. HIV) to another. Leveraging resources from infectious disease infrastructure is particularly important in light of the fact that a large percentage of cancers in developing countries are infection-associated. For example, allied health worker training could be enhanced by partnerships between neighboring institutes or countries to help address the challenge of critical shortages of health workforce and access to specialized training that is required for optimal multidisciplinary management of cancer patients.

Many partnerships between LMICs already exist in the general areas of biotechnology and health, such as partnerships between Cuba and Brazil, South Africa and India and Brazil, and partnerships between countries in Africa. In large part, these partnerships tend to have a commercial focus including development of novel anticancer agents [14]. Some trilateral collaborations that include so-called “South-South” partnerships focus on developing scientific and educational environments in LMICs such as funding of innovative research through the International Center for Genetic Engineering and Biotechnology. In addition, South-South collaborations have seen much success in the development of vaccines. Given the development of HBV and HPV vaccines to prevent cancer and the relative proportion of infection attributable cancers in the LMICs, it is not difficult to imagine how beneficial the integration of South-South collaborative partnerships into cancer centers in LMICs can be in strengthening cancer control and prevention.

To ensure the highest likelihood of success for new and continued cancer center success in LMICs, a detailed understanding of how challenges were addressed previously is essential. Data on resources, capacities, priorities, barriers, and the unique circumstances of local institutes in LMICs that prevent, diagnose, treat, manage, and research cancers is needed for those seeking to adapt successful models. Analysis of that data should answer a number of key questions including: What are the critical factors for success? What common (and less obvious) pitfalls can be avoided? How do you ensure equity and mutual benefit within partnerships? How do you build trust within partnerships? How can evidenced-based solutions be best adapted to the local context? What standard operating procedures are prudent to have in place?

The *Cancer Centers in Low- and Middle-Income Countries* series is intended to make information and lessons-learned about successful cancer centers in LMICs more broadly available for those seeking to replicate successful models and to help re-invigorate efforts to build effective and sustainable partnerships. The series will provide a platform to showcase detailed features of institutes from LMICs and their collaborations including: (1) cancer center activities and resources for prevention, diagnosis, treatment and palliative care (available facilities, health care worker resources, outreach programs, collaborations, research projects, oncology-related training facilities, etc.); (2) funding support from local government, foundations, or privately borne by patients; (3) key features of the cancer center and its partnerships that contribute to its success; (4) challenges, both universal and setting-specific, and how they are addressed; (5) policy and practice implications including discussion of what could be adapted by others, what gaps need to be addressed, and what differences might arise in other settings. With detailed information in hand about the lessons learned in developing successful cancer center programs in LMICs, those who aim to address the growing burden of cancer in the developing world will be much better equipped to do so.

The authors’ opinions and conclusions cannot be construed as official policy statements of NIH, HHS, or the United States government.

## References

[B1] Ferlay J Shin HR Bray F Forman D Mathers C Parkin DM GLOBOCAN 2008 v2.0, Cancer Incidence and Mortality Worldwide: IARC CancerBase No. 10 [Internet] 2010 Lyon, France: International Agency for Research on Cancer Accessed on Oct 18, 2013. http://globocan.iarc.fr

[B2] WHO Cancer Fact Sheet No. 297 http://www.who.int/mediacentre/factsheets/fs297/en/index.html

[B3] de Martel C Ferlay J Franceschi S Vignat J Bray F Forman D Plummer M Global burden of cancers attributable to infections in 2008: a review and synthetic analysis Lancet Oncol 2012 8 607 615 10.1016/S1470-2045(12)70137-7 22575588

[B4] Tierney WM Nyandiko WN Siika AM Wools-Kaloustian K Sidle JE Kiplagat J Bell A Inui TS "These are good problems to have…": establishing a collaborative research partnership in East Africa J Gen Intern Med 2013 8 S625 S638 10.1007/s11606-013-2459-4 23797916 PMC3744278

[B5] Moormann A Skiles J Koros E Asirwa FC Busakhala N Loehrer P Mentoring future Kenyan oncology researchers Infect Agent Cancer 2013 8 40 10.1186/1750-9378-8-40 24099090 PMC3851568

[B6] Strother RM Fitch M Kamau P Beattie K Boudreau A Busakhalla N Loehrer PJ Building cancer nursing skills in a resource-constrained government hospital Support Care Cancer 2012 8 2211 2215 10.1007/s00520-012-1482-z 22565597

[B7] Strother RM Rao KV Gregory KM Jakait B Busakhala N Schellhase E Pastakia S Krzyzanowska M Loehrer PJ The oncology pharmacy in cancer care delivery in a resource-constrained setting in western Kenya J Oncol Pharm Pract 2012 8 406 416 10.1177/1078155211434852 22249828

[B8] Strother RM Gregory KM Pastakia SD Were P Tenge C Busakhala N Jakait B Schellhase EM Rosmarin AG Loehrer PJ Retrospective analysis of the efficacy of gemcitabine for previously treated AIDS-associated Kaposi's sarcoma in western Kenya Oncology 2010 8 5 11 10.1159/000292356 20215784

[B9] Ting J Mugo N Kwatampora J Hill C Chitwa M Patel S Gakure H Kimani J Schoenbach VJ Poole C Smith JS High-risk human papillomavirus messenger RNA testing in physician- and self-collected specimens for cervical lesion detection in high-risk women, Kenya Sex Transm Dis 2013 8 584 589 10.1097/OLQ.0b013e31828e5a91 23965776

[B10] Becker-Dreps S Otieno WA Brewer NT Agot K Smith JS HPV vaccine acceptability among Kenyan women Vaccine 2010 8 4864 4867 10.1016/j.vaccine.2010.05.034 20566394 PMC2906622

[B11] Coghill AE Newcomb PA Madeleine MM Richardson BA Mutyaba I Okuku F Phipps W Wabinga H Orem J Casper C Contribution of HIV infection to mortality among cancer patients in Uganda AIDS 2013 8 2933 2942 10.1097/01.aids.0000433236.55937.cb PMC4319357 23921614

[B12] Phipps W Ssewankambo F Nguyen H Saracino M Wald A Corey L Orem J Kambugu A Casper C Gender differences in clinical presentation and outcomes of epidemic Kaposi sarcoma in Uganda PLoS One 2010 8 e13936 10.1371/journal.pone.0013936 21103057 PMC2980479

[B13] Hassan MH Building capacity in the life sciences in the developing world Cell 2007 8 433 436 10.1016/j.cell.2007.10.020 17981107

[B14] Saenz TW Thorsteinsdottir H de Souza MC Cuba and Brazil: an important example of South-South collaboration in health biotechnology MEDICC Rev 2010 8 32 35 20697336 10.37757/MR2010.V12.N3.8

